# Human oesophageal adenocarcinoma cell lines JROECL 47 and JROECL 50 are admixtures of the human colon carcinoma cell line HCT 116

**DOI:** 10.1054/bjoc.1999.1170

**Published:** 2000-04-03

**Authors:** B P L Wijnhoven, M G J Tilanus, A G Morris, S J Darnton, H W Tilanus, W N M Dinjens

**Affiliations:** The Rotterdam Oesophageal Tumour Study Group, Erasmus University Medical Centre Rotterdam, Rotterdam, The Netherlands; Department of Surgery, University Hospital Dijkzigt, Dr Molewaterplein 40, Rotterdam, GD 3015, The Netherlands; Department of Pathology, University Medical Center, Heidelberglaan 100, Utrecht, CX 3584, The Netherlands; Department of Biological Sciences, University of Warwick, Gibbert Hill Road, Coventry, CV4 7AL, UK; Department of Thoracic Surgery, Birmingham Heartlands Hospital, Bordesley Green East, Birmingham, B9 5SS, UK; Department of Pathology, Josephine Nefkens Institute, PO Box 1738, Rotterdam, DR 3000, The Netherlands

**Keywords:** adenocarcinoma, oesophagus, colon, cell lines, admixtures

## Abstract

In two recently described human oesophageal adenocarcinoma cell lines JROECL 47 and JROECL 50, derived from one tumour, we detected identical E-cadherin and β-catenin gene mutations as in colon carcinoma cell line HCT 116. We demonstrate by HLA-typing, mutation analysis and microsatellite analysis that cell lines JROECL 47 and JROECL 50 are admixtures of the human colon adenocarcinoma cell line HCT 116. © 2000 Cancer Research Campaign


*British Journal of Cancer* (2000) **82**(9), 1510–1512
© 2000 Cancer Research Campaign
DOI: 10.1054/ bjoc.1999.1170, available online at http://www.idealibrary.com on IDEAL®

### Human oesophageal adenocarcinoma cell lines JROECL 47 and JROECL 50 are admixtures of the human colon carcinoma cell line HCT 116

**BPL Wijnhoven[1,2], MGJ Tilanus[3], AG Morris[4], SJ Darnton[5], HW Tilanus[1,2] and WNM Dinjens[1,6]**

[1]The Rotterdam Oesophageal Tumour Study Group, Erasmus University Medical Centre Rotterdam, Rotterdam, The Netherlands; [2]Department of Surgery, University Hospital Dijkzigt, Dr Molewaterplein 40, 3015 GD Rotterdam, The Netherlands; [3]Department of Pathology, University Medical Center, Heidelberglaan 100, 3584 CX Utrecht, The Netherlands; [4]Department of Biological Sciences, University of Warwick, Gibbert Hill Road, Coventry CV4 7AL, UK; [5]Department of Thoracic Surgery, Birmingham Heartlands Hospital, Bordesley Green East, Birmingham B9 5SS, UK; [6]Department of Pathology, Josephine Nefkens Institute, PO Box 1738, 3000 DR Rotterdam, The Netherlands

**Summary** In two recently described human oesophageal adenocarcinoma cell lines JROECL 47 and JROECL 50, derived from one tumour, we detected identical E-cadherin and β-catenin gene mutations as in colon carcinoma cell line HCT 116. We demonstrate by HLA-typing, mutation analysis and microsatellite analysis that cell lines JROECL 47 and JROECL 50 are admixtures of the human colon adenocarcinoma cell line HCT 116. © 2000 Cancer Research Campaign

**Keywords**: adenocarcinoma, oesophagus, colon, cell lines, admixtures

Recently, four human oesophageal and gastric cardia adenocarcinoma cell lines were established (Rockett et al, 1997). These cell lines were included in two studies on E-cadherin and β-catenin gene mutations in adenocarcinomas of the oesophagus (Wijnhoven et al, 1999; Wijnhoven et al, 2000). Cell lines JROECL 47 and JROECL 50, derived from one tumour, harbour E-cadherin and β-catenin gene mutations. These mutations could not be detected in the primary tumour from which the cell lines were established. Recently, identical E-cadherin and β-catenin gene mutations have been described in the human colon tumour cell line HCT 116, established in 1981 (Brattain et al, 1981; Efstathiou et al, 1999; Ilyas et al, 1997). These results prompted us to investigate the derivation of the cell lines JROECL 47 and JROECL 50 by HLA-typing, mutation analyses, microsatellite allelotyping and microsatellite instability (MSI) analysis.

### MATERIALS AND METHODS

### Cell lines, primary tumour, xenografts and DNA isolation

Cell lines JROECL 47 (passage 16) and JROECL 50 (passage 10) were obtained from the European Collection of Cell Cultures (ECACC). From cell lines JROECL 47 and JROECL 50 the early passages, before submission of these cell lines to the ECACC (passages 2 and 4, respectively) were also investigated. These early passages were a gift from Dr AG Morris, University of Warwick, Coventry, UK. Sections from the original paraffin tissue blocks of the patient's oesophageal tumour, from which the cell lines JROECL 47 and 50 were presumably derived, were gifted by Dr SJ Darnton, Birmingham Heartlands Hospital, Birmingham, UK. Colon cancer cell line HCT 116 was a generous gift from Dr P van der Saag, Hubrecht Laboratory, Utrecht, the Netherlands. Cells were cultured under standard conditions in RPMI 1640 supplemented with 10% FCS.

To study the histological characteristics, $5 \times 10^6$ trypsinized tumour cells from cell lines JROECL 47 and 50 (passages 16 and 10, respectively) and HCT 116 were injected subcutaneously in female NMRI nude mice. Xenografts were removed and routinely processed for histological examination. The animal experiments were licensed and done in accordance with approved protocols by the Erasmus University Medical Centre, Rotterdam, The Netherlands.

DNA was isolated by standard proteinase K digestion and phenol extraction from the cultured cell lines and from the tissue block of the original oesophageal tumour, from which cell lines JROECL 47 and JROECL 50 were presumably established.

### HLA typing

HLA-DRB1 typing was performed on cell lines JROECL 47 and 50, cell line HCT116 and the tissue blocks of the original tumour, as described (McGinnis et al, 1995). The polymorphic exon 2 was amplified and subsequently sequenced on an ABI373 automated sequencer (Perkin Elmer, Foster City, USA). HLA-DRB allele assignment was established by comparing the sequences obtained to the HLA-allele database similar to HLA-DPB allele assignment (Versluis et al, 1993).

### Mutation analyses

Cell line HCT 116 has heterozygous mutations in the E-cadherin gene (codon 120; exon 3), the β-catenin gene (codon 45; exon 3) and the K-*ras* gene (codon 13; exon 1) (Buard et al, 1996;

*Received 30 September 1999*
*Revised 14 December 1999*
*Accepted 20 December 1999*

*Correspondence to:* WNM Dinjens

Efstathiou et al, 1999; Ilyas et al, 1997). PCR-SSCP was performed to detect these mutations, as described (Buard et al, 1996; Fukuchi et al, 1998; Wijnhoven et al, 1999). Samples with aberrant migrating bands were reamplified, cloned and sequenced.

### Microsatellite analyses

Nine polymorphic dinucleotide repeat markers: D8S136, D8S133, D9S161, D9S156, D16S265, D14S292, D14S977, D17S786 and CHRNB1 were investigated by radioactive PCR as described previously (Trapman et al, 1994).

Because HCT 116 is reported to have the microsatellite instable (MSI) phenotype (Hoang et al, 1997), MSI markers BAT26, BAT40 and BAT-RII were also investigated (Grady et al, 1998).

### RESULTS AND DISCUSSION

To date, only very few in vitro growing human oesophageal adenocarcinoma cell lines are known. The availability of these cell lines is of great value to study the biology and the genetic alterations in these poorly-understood cancers, which show a dramatic increase in incidence over the past decades (McKinney et al, 1995). Recently, four such cell lines were established (Rockett et al, 1997). Here we report that two of these cell lines, JROECL 47 and JROECL 50 are in fact admixtures of the human colon cancer cell line HCT 116.

In all experiments identical results were obtained for the early and late passages of cell lines JROECL 47 and JROECL 50. In cell culture JROECL 47, JROECL 50 and HCT 116 have the same morphology with spindle shaped cells and similar growth rates. Xenografting of these three cell lines resulted in undifferentiated solid tumours, without glandular differentiation (results not shown). HLA typing revealed that cell lines JROECL 47, JROECL 50 and HCT 116 all have the same HLA-DR allele DRB1 *03011/1102, which is different from the original primary oesophageal tumour from which the cell lines JROECL 47 and 50 were presumably established: DRB1 *08032/04011. An example of the difference between the cell lines and the original primary tumour is shown by a characteristic sequence of exon 2 of the primary tumour and the cell lines (Figure 1). The frequency of the patient primary tumour allele combination DRB1 *08032/04011 in the population is less than 0.0041 compared to the frequency of 0.0098 of the allele combination DRB1*03011/1102 of the cell lines (Schipper et al, 1996). Furthermore, PCR-SSCP analyses of exon 3 of the E-cadherin gene, exon 3 of the β-catenin gene and codon 12/13 of the K-*ras* gene showed an identical, aberrant mobility pattern in all three cell lines (Figure 2). Upon sequencing of the samples with aberrant migration patterns, the reported mutations in all three genes were confirmed (results not shown) (Buard et al, 1996; Efstathiou et al, 1999; Ilyas et al, 1997).

Allelotyping, however, showed different allele sizes between the three cell lines with 7/9 polymorphic markers, indicating a different origin of the cell lines (Figure 3A). With two markers the allele patterns were identical between the cell lines. But all three MSI markers demonstrated pronounced microsatellite instability with different allele sizes in the three cell lines. Figure 3B represents an example of MSI in the three cell lines as demonstrated by BAT26. Indeed, HCT 116 has been reported to have an extremely microsatellite instable phenotype (Oki et al, 1999). Obviously, separate cultures of HCT 116 resulted in different microsatellite alterations. Therefore, microsatellite analysis is not appropriate for allelotyping MSI cell lines.

Our assumption that cell lines JROECL 47 and 50 are admixtures of HCT 116 was confirmed by the ECACC with DNA fingerprinting (personal communication). Therefore, we conclude that cell lines JROECL 47 and JROECL 50 are not human oesophageal adenocarcinoma cell lines, but are admixtures of the human colon adenocarcinoma cell line HCT 116.

```

                                      1111111    1122222222   222

                   2333333345   7990046777   7900111122   355

                   8012567867   7472909013   4978012801   078

                   ----------   ----------   ----------   ---

Primary tumour     TCTCCGGGTT   TTAATAAGCC   CAAGCAGCCT   CGT  DRB1* 08032

                   CGGTAACACC   ..C...GAT.   .CGCG.A.GC   ...  DRB1* 04011

Cell lines         ......TCC.   AC.GATGAT.   .CGCG.AG.G   ATG  DRB1* 03011

                   ......TCC.   .....TGATA   G...GA.GC    .TG  DRB1* 1102

```

**Figure 1** HLA-typing of the original primary tumour and the cell lines (JROECL 47 and 50 and HCT 116). The polymorphic positions of HLA-DRB1 exon 2 are shown in vertical orientation. Dots indicate identity to the nucleotide of the DRB1*08032 allele. Numbers are the polymorphic positions of nucleotides in exon 2 shown in vertical orientation (position 28, 30, … 258). The polymorphic positions of the original primary tumour (DRB1*08032/04011) have been compared with the cell lines. The alleles present in the primary tumour do not exist in the cell lines.

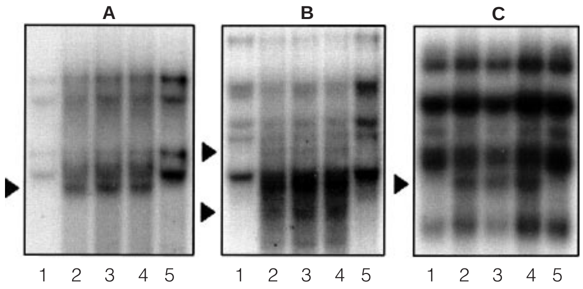

**Figure 2** PCR-SSCP analysis of the E-cadherin gene exon 3 (**A**), β-catenin gene exon 3 (**B**) and K-*ras* gene codon 12/13 (**C**). Lanes 1 and 5, normal, non-mutated control DNA from one individual; lane 2, JROECL 47; lane 3, JROECL 50 and lane 4, HCT 116. Note the same aberrant migration patterns in all 3 cell lines (arrowheads), as compared to normal control DNA.

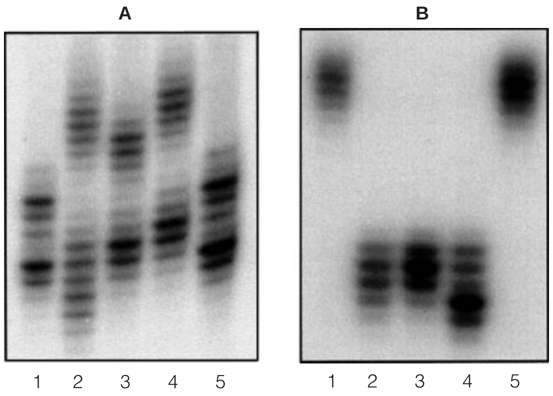

**Figure 3** Allelotyping with polymorphic marker CHRNB1 (**A**) and microsatellite analysis with BAT26 (**B**). Lanes 1 and 5, normal control DNA from one individual; lane 2, JROECL 47; lane 3, JROECL 50 and lane 4, HCT 116. Note the different allele sizes in the three cell lines, indicating different origins (**A**). Note the alteration in mononucleotide repeat size in the cell line DNAs compared to normal, control DNA, indicating MSI (**B**).

© 2000 Cancer Research Campaign

Furthermore, allelotyping of cell lines by microsatellite analysis can be hampered by MSI.

### ACKNOWLEDGEMENTS

The authors thank H Sleddens, N Groen, AW van der Zwan, E Rozemuller and NJ de Both for technical assistance.

### REFERENCES

Brattain MG, Fine WD, Khaled FM, Thompson J and Brattain DE (1981) Heterogeneity of malignant cells from a human colonic carcinoma. *Cancer Res* **41**: 1751–1756

Buard A, Zipfel PA, Frey RS and Mulder KM (1996) Maintenance of growth factor signaling through *Ras* in human colon carcinoma cells containing K-*ras* mutations. *Int J Cancer* **67**: 539–546

Efstathiou JA, Liu D, Wheeler JM, Kim HC, Beck NE, Ilyas M, Karayiannakis AJ, Mortensen NJ, Kmiot W, Playford RJ, Pignatelli M and Bodmer WF (1999) Mutated epithelial cadherin is associated with increased tumorigenicity and loss of adhesion and of responsiveness to the motogenic trefoil factor 2 in colon carcinoma cells. *Proc Natl Acad Sci USA* **96**: 2316–2321

Fukuchi T, Sakamoto M, Tsuda H, Maruyama K, Nozawa S and Hirohashi S (1998) Beta-catenin mutation in carcinoma of the uterine endometrium. *Cancer Res* **58**: 3526–3528

Grady WM, Rajput A, Myeroff L, Liu DF, Kwon K, Willis J and Markowitz S (1998) Mutation of the type II transforming growth factor-beta receptor is coincident with the transformation of human colon adenomas to malignant carcinomas. *Cancer Res* **58**: 3101–3104

Hoang JM, Cottu PH, Thuille B, Salmon RJ, Thomas G and Hamelin R (1997) BAT-26, an indicator of the replication error phenotype in colorectal cancers and cell lines. *Cancer Res* **57**: 300–303

Ilyas M, Tomlinson IP, Rowan A, Pignatelli M and Bodmer WF (1997) Beta-catenin mutations in cell lines established from human colorectal cancers. *Proc Natl Acad Sci USA* **94**: 10330–10334

McGinnis MD, Conrad MP, Bouwens AG, Tilanus MG and Kronick MN (1995) Automated, solid-phase sequencing of DRB region genes using T7 sequencing chemistry and dye-labeled primers. *Tissue Antigens* **46**: 173–179

McKinney A, Sharp L, Macfarlane GJ and Muir CS (1995) Oesophageal and gastric cancer in Scotland 1960–90. *Br J Cancer* **71**: 411–415

Oki E, Oda S, Maehara Y and Sugimachi K (1999) Mutated gene-specific phenotypes of dinucleotide repeat instability in human colorectal carcinoma cell lines deficient in DNA mismatch repair. *Oncogene* **18**: 2143–2147

Rockett JC, Larkin K, Darnton SJ, Morris AG and Matthews HR (1997) Five newly established oesophageal carcinoma cell lines: phenotypic and immunological characterization. *Br J Cancer* **75**: 258–263

Schipper RF, Schreuder GM, D'Amaro J and Oudshoorn M (1996) HLA gene and haplotype frequencies in Dutch blood donors. *Tissue Antigens* **48**: 562–574

Trapman J, Sleddens HF, van der Weiden MM, Dinjens WN, Konig JJ, Schroder FH, Faber PW and Bosman FT (1994) Loss of heterozygosity of chromosome 8 microsatellite loci implicates a candidate tumor suppressor gene between the loci D8S87 and D8S133 in human prostate cancer. *Cancer Res* **54**: 6061–6064

Versluis LF, Rozemuller E, Tonks S, Marsh SG, Bouwens AG, Bodmer JG and Tilanus MG (1993) High-resolution HLA-DPB typing based upon computerized analysis of data obtained by fluorescent sequencing of the amplified polymorphic exon 2. *Hum Immunol* **38**: 277–283

Wijnhoven BP, de Both NJ, van Dekken H, Tilanus HW and Dinjens WN (1999) E-cadherin gene mutations are rare in adenocarcinomas of the oesophagus. *Br J Cancer* **80**: 1652–1657

Wijnhoven BPL, Nollet F, de Both NJ, Tilanus HW and Dinjens WNM (2000). Genetic alterations involving exon 3 of the beta-catenin gene do not play a role in adenocarcinomas of the oesophagus. *Int J Cancer* (in press)

 *© 2000 Cancer Research Campaign*

## References

[PDF_00225] Brattain M. G., Fine W. D., Khaled F. M., Thompson J., Brattain D. E. (1981). Heterogeneity of malignant cells from a human colonic carcinoma.. Cancer Res.

[PDF_00228] Buard A., Zipfel P. A., Frey R. S., Mulder K. M. (1996). Maintenance of growth factor signaling through Ras in human colon carcinoma cells containing K-ras mutations.. Int J Cancer.

[PDF_00231] Efstathiou J. A., Liu D., Wheeler J. M., Kim H. C., Beck N. E., Ilyas M., Karayiannakis A. J., Mortensen N. J., Kmiot W., Playford R. J. (1999). Mutated epithelial cadherin is associated with increased tumorigenicity and loss of adhesion and of responsiveness to the motogenic trefoil factor 2 in colon carcinoma cells.. Proc Natl Acad Sci U S A.

[PDF_00236] Fukuchi T., Sakamoto M., Tsuda H., Maruyama K., Nozawa S., Hirohashi S. (1998). Beta-catenin mutation in carcinoma of the uterine endometrium.. Cancer Res.

[PDF_00239] Grady W. M., Rajput A., Myeroff L., Liu D. F., Kwon K., Willis J., Markowitz S. (1998). Mutation of the type II transforming growth factor-beta receptor is coincident with the transformation of human colon adenomas to malignant carcinomas.. Cancer Res.

[PDF_00243] Hoang J. M., Cottu P. H., Thuille B., Salmon R. J., Thomas G., Hamelin R. (1997). BAT-26, an indicator of the replication error phenotype in colorectal cancers and cell lines.. Cancer Res.

[PDF_00246] Ilyas M., Tomlinson I. P., Rowan A., Pignatelli M., Bodmer W. F. (1997). Beta-catenin mutations in cell lines established from human colorectal cancers.. Proc Natl Acad Sci U S A.

[PDF_00213] Lane J. R. (1872). Clinical Lecture on Injuries from Lightning.. Br Med J.

[PDF_00249] McGinnis M. D., Conrad M. P., Bouwens A. G., Tilanus M. G., Kronick M. N. (1995). Automated, solid-phase sequencing of DRB region genes using T7 sequencing chemistry and dye-labeled primers.. Tissue Antigens.

[PDF_00252] McKinney A., Sharp L., Macfarlane G. J., Muir C. S. (1995). Oesophageal and gastric cancer in Scotland 1960-90.. Br J Cancer.

[PDF_00254] Oki E., Oda S., Maehara Y., Sugimachi K. (1999). Mutated gene-specific phenotypes of dinucleotide repeat instability in human colorectal carcinoma cell lines deficient in DNA mismatch repair.. Oncogene.

[PDF_00257] Rockett J. C., Larkin K., Darnton S. J., Morris A. G., Matthews H. R. (1997). Five newly established oesophageal carcinoma cell lines: phenotypic and immunological characterization.. Br J Cancer.

[PDF_00260] Schipper R. F., Schreuder G. M., D'Amaro J., Oudshoorn M. (1996). HLA gene and haplotype frequencies in Dutch blood donors.. Tissue Antigens.

[PDF_00263] Trapman J., Sleddens H. F., van der Weiden M. M., Dinjens W. N., Konig J. J., Schroder F. H., Faber P. W., Bosman F. T. (1994). Loss of heterozygosity of chromosome 8 microsatellite loci implicates a candidate tumor suppressor gene between the loci D8S87 and D8S133 in human prostate cancer.. Cancer Res.

[PDF_00268] Versluis L. F., Rozemuller E., Tonks S., Marsh S. G., Bouwens A. G., Bodmer J. G., Tilanus M. G. (1993). High-resolution HLA-DPB typing based upon computerized analysis of data obtained by fluorescent sequencing of the amplified polymorphic exon 2.. Hum Immunol.

[PDF_00272] Wijnhoven B. P., de Both N. J., van Dekken H., Tilanus H. W., Dinjens W. N. (1999). E-cadherin gene mutations are rare in adenocarcinomas of the oesophagus.. Br J Cancer.

